# Preparation of UiO-66 MOF-Bonded Porous-Layer Open-Tubular Columns Using an In Situ Growth Approach for Gas Chromatography

**DOI:** 10.3390/molecules29112505

**Published:** 2024-05-25

**Authors:** Khadejah D. Otaif, Ahmed-Yacine Badjah-Hadj-Ahmed, Zeid Abdullah ALOthman

**Affiliations:** 1 Department of Chemistry, College of Science, Jazan University, Jazan 82843, Saudi Arabia; 2 Advanced Materials Research Chair, Department of Chemistry, College of Science, King Saud University, Riyadh 11451, Saudi Arabia; ybadjah@ksu.edu.sa; 3 Department of Chemistry, College of Science, King Saud University, P.O. Box 2455, Riyadh 11451, Saudi Arabia; zaothman@ksu.edu.sa

**Keywords:** metal–organic frameworks, in situ growth, UiO-66, fast separation, gas chromatography

## Abstract

The thermally stable zirconium-based MOF, UiO-66, was employed for the preparation of bonded porous-layer open-tubular (PLOT) GC columns. The synthesis included the in situ growth of the UiO-66 film on the inner wall of the capillary through a one-step solvothermal procedure. SEM–EDX analysis revealed the formation of a thin, continuous, uniform, and compact layer of UiO-66 polycrystals on the functionalized inner wall of the column. The average polarity (Δ*I*_av_ = 700) and the McReynolds constants reflected the polar nature of the UiO-66 stationary phase. Several mixtures of small organic compounds and real samples were used to evaluate the separation performance of the fabricated columns. Linear alkanes from *n*-pentane to *n*-decane were baseline separated within 1.35 min. Also, a series of six *n*-alkylbenzenes (C_3_–C_8_) were separated within 3 min with a minimum resolution of 3.09, whereas monohalobenzene mixtures were separated at 220 °C within 14s. UiO-66 PLOT columns are ideally suited for the isothermal separation of chlorobenzene structural isomers at 210 °C within 45 s with R_s_ ≥ 1.37. The prepared column featured outstanding thermal stability (up to 450 °C) without any observed bleeding or significant impact on its performance. This feature enabled the analysis of various petroleum-based samples.

## 1. Introduction

Chromatography has become the dominant analytical tool for separating, identifying, and quantifying a wide range of analytes. The most important part of the chromatographic system is the separation column, which is generally classified into two groups, namely, open-tubular (OT) and packed columns. Compared to classically packed columns, OT columns offer higher separation efficiency, better resolution, more defined peaks, improved sensitivity, higher permeability, and shorter run times. Additionally, the small capillary internal diameter is resource-efficient, as it requires less stationary phase packing material, smaller sample volume, and lower mobile phase flow rates. Stationary phases can either be physically coated or chemically bonded to the capillary inner wall. They are classified into three major types, namely, inorganic, organic, and inorganic–organic hybrid materials [1,2].

Metal–organic frameworks (MOFs) are the newest type of stationary phases commonly employed for chromatographic columns. They are a class of promising porous organic–inorganic hybrid materials first introduced by Yaghi et al. [3] in 1995. MOFs consist of metal ions or clusters linked coordinatively to organic ligands. These linkers could be di- or polyfunctional carboxylates, sulfonates, phosphonates, or heterocyclic compounds. The metal clusters, which are termed secondary building units (SBUs), are used for classification purposes. MOFs exhibit unique properties that make them desirable substrates for fabricating chromatographic columns, including high porosity, large surface area, and well-defined, homogenous pores. MOFs have become popular in research as they exhibit unprecedented structural designability and diversity [4,5,6].

Various methods are used to synthesize MOFs as the selected method significantly affects the reaction yield, particle size, size distribution, and surface morphology of the associated MOF or the subsequent reaction process. Thus, the primary determinant for choosing the appropriate method of synthesis depends on the required properties of the product MOF. Common synthetic methods include (i) slow evaporation, (ii) hydro/solvothermal, (iii) microwave-assisted, (vi) electrochemical, (v) mechanochemical, and (vi) sonochemical synthesis [7,8]. Although MOFs are frequently isolated as powders, they can also be shaped into several forms to serve a specific application, and are commonly found as granules, monoliths, pellets, thin films, foams, and hollow structures [9]. MOF films are often employed for several interesting applications, namely, for the production of semiconductors [10] and sensors [11], as well as for gas separation and storage [12,13], reaction catalysis [14], and chromatography [15].

In chromatographic applications, customized MOF stationary phases can be prepared using different strategies. Powder MOFs can be pre-synthesized and then packed into the columns to produce “particle-packed columns” [16,17] or incorporated into polymer monoliths to fabricate “monolithic columns” [18,19]. For porous-layer open-tubular (PLOT) capillary columns, the pre-synthesized MOF particles can be physically deposited onto the fused-silica capillary inner walls by injecting the MOF suspension and then evaporating the solvent [20,21]. Despite the fast and straightforward procedure of physical deposition, this process is not selective, and it is difficult to form uniform layers [22]. Moreover, the weakness of the physical interactions between the MOF particles and the column walls reduces the stability of the chromatographic column and causes column bleeding over time, especially at elevated temperatures. However, bonded PLOT columns suppress stationary phase bleeding by forming strong chemical bonds between the coated material and the column walls [23]. The fabrication of highly stable MOF-based PLOT columns can be achieved by growing the MOF films in situ on chemically modified fused-silica capillary walls as polycrystalline films or surface-mounted metal–organic frameworks (SURMOFs).

Regarding the chemical modification approach, the substrate (i.e., the fused-silica capillary wall in this case) can be functionalized with suitable functional groups (e.g., –OH, –COOH, –NH_2_, etc.) that coordinate with the MOF metal ions to form a self-assembled monolayer (SAM). SAMs serve as adhesive surfaces and templates for orienting the growth of MOF films [24]. For instance, silane coupling agents, namely, 3-aminopropyltriethoxysilane (APTES) [25,26], 3-aminopropyltrimethoxysilane (APTMS) [27], and (3-glycidoxypropyl)triethoxysilane (GLYMO) [28], are frequently used to form strong chemical bonds between the fused-silica capillary wall surfaces and the respective MOF films. This stepwise procedure is tedious and time-consuming, even with the high structural quality and homogeneity of the SURMOFs. Additionally, several preparation conditions require systematic optimization to ensure the formation of the relevant SURMOFs. On the other hand, in situ crystallization from a solvothermal mother solution is a straightforward, one-step method for forming MOF films directly inside the capillary, with the added benefit of employing the same solvothermal synthesis conditions for the formation of MOF powders [22]. Moreover, rapid heating techniques can also be used to reduce the time for the conventional solvothermal synthesis procedure.

Chen et al. [29] reported the first application of MOFs as packing in GC. Here, the MOF-508 [Zn(BDC)(4,4′-Bipy)_0.5_(DMF)(H_2_O)_0.5_] single crystals used showed thermal stability up to 360 °C and were capable of efficiently separating linear alkanes from branched ones. Various MOF-based stationary phases have also been employed for GC applications, such as ZIF-8 [30], IRMOF-3 [21], ZIF-90 [20], HKUST-1 [31], MOF-5 [23], MIL-100(Fe), (Cr) [32], CPL-1 [17], and chiral MOF [In_3_O(obb)_3_(HCO_2_)(H_2_O)] [33].

University of Oslo 66 (abbreviated UiO-66) is a zirconium-based MOF that consists of a Zr_6_O_4_(OH)_4_ core connected to twelve 1,4-benzene-dicarboxylate (BDC) linkers to form the Zr_6_O_4_(OH)_4_(CO_2_)_12_ cluster. It possesses two types of rigid cubic 3D cavities, namely, an octahedral cavity with a diameter of 11 Å and a tetrahedral cavity with a diameter of 8 Å. The microporous triangular windows of the cavities have a diameter range of 5–7 Å that provides access to the cavities. UiO-66 exhibits very high thermal stability (up to 540 °C), good chemical resistance against various solvents (e.g., water, acetone, benzene, and DCM), excellent mechanical stability (>10 tons/cm^2^), and high adsorption capacity [34]. These distinctive properties make UiO-66 MOF ideally suited as a stationary phase for gas chromatography. Chang et al. prepared a UiO-66 physically coated open-tubular column to investigate the reverse shape selectivity and molecular sieving effect of the prepared column during the separation of alkane isomers and benzene compounds [35]. Nowadays, bonded PLOTs have become convenient and suitable for a wide range of GC applications. As discussed above, chemical bonding between the stationary phase and the column wall enhances column stability and suppresses column bleeding. This leads to a longer life of the column, better sensitivity (i.e., improved signal-to-noise ratio), and higher reproducibility. Additionally, it protects the instrumentation (especially the detectors) from contamination. There are no studies to date on the preparation and application of a UiO-66-bonded PLOT column for GC applications.

The main goal of the current work was to fabricate novel and stable UiO-66-based, chemically bonded PLOT GC columns using a straightforward, one-step in situ solvothermal procedure. We also investigated the separation performance of the prepared columns by analyzing several mixtures of small organic compounds and evaluated the prepared columns’ ability to achieve fast GC analysis.

## 2. Results and Discussion

### 2.1. Characterization of the UiO-66-Bonded PLOT Column

**Figure 1:**
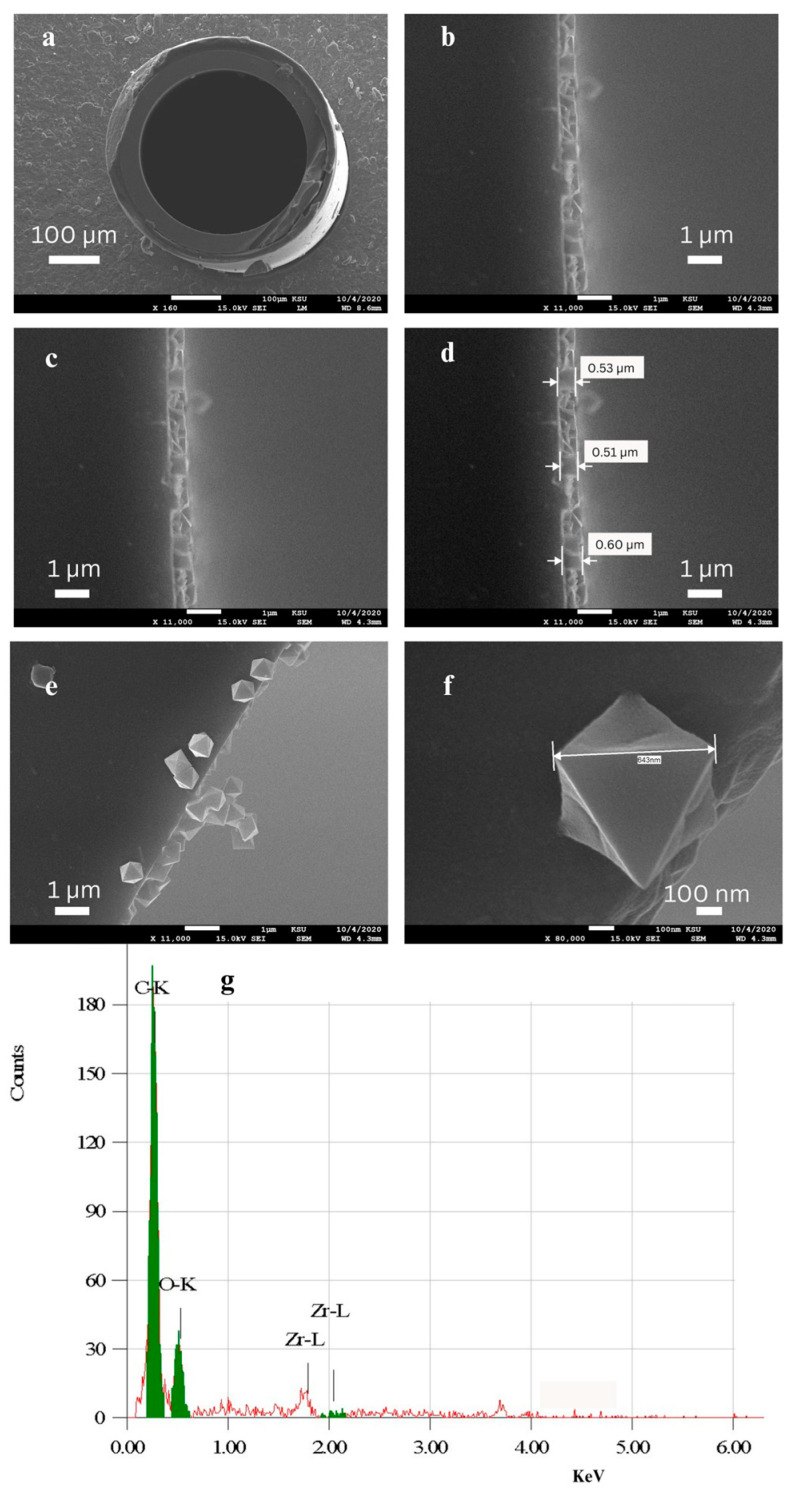
SEM images of the cross-sections of (**a**) the bare capillary; (**b**–**f**) the UiO-66 column; and (**g**) the EDX graph of the UiO-66 column.

SEM images were obtained for the bare fused-silica capillary and for the prepared UiO-66 PLOT column to verify the formation of the UiO-66 film on the column’s inner wall and to determine the morphology of the grown film. Figure 1a presents the open path and the smooth inner wall of the capillary, which reflects the low thickness of the MOF layers. Figure 1b–d show the formation of a thin, continuous, uniform, and compact layer of UiO-66 polycrystals on the functionalized inner wall of the column with a thickness of about 0.5 µm. Regarding the surface morphology, the SEM micrographs in Figure 1e,f clearly show the high-quality octahedral UiO-66 nanocrystals. The scattered crystallites could result from the rupture of the MOF layer following the cutting of the capillary tube. In addition, the characterization of the MOF layer via energy-dispersive X-ray spectroscopy (EDX) confirmed the presence of the expected main elements. EDX analysis (Figure 1g) supported the SEM results and gave further indication of the formation of the UiO-66 stationary phase layer.

### 2.2. Polarity of the UiO-66 Stationary Phase

The McReynolds constants were calculated with two main goals in mind. The first was to quantify the polarity of the prepared UiO-66 PLOT column and to compare it to that of other GC stationary phases. The second goal was to evaluate the extent of the intermolecular interactions between the probes and the UiO-66 stationary phase, which may help in understanding its retention mechanism.

The calculated retention indices of the probes on the squalane and UiO-66 columns are shown in Table 1. The McReynolds coefficients of the studied column, UiO-66 PLOT, and other GC stationary phases are listed in Table 2 in the order of increasing polarity. The high Δ*I* values for the Y’–S’ probes in UiO-66 are indicative of extensive interactions with these chemical families and reflect the extremely polar nature of the UiO-66 stationary phase. The low value for the benzene parameter, X’, indicates that UiO-66 weakly interacts with aromatics compared to the non-polar TR-1 reference column. When the average polarity of the listed stationary phases is compared, it can be seen that the UiO-66 column corresponds to the most polar material. This strong polar nature can be attributed to the polar functionalities (i.e., –OH and –COOH) within the UiO-66 framework.

Moreover, the presence of open metal sites, such as the electron-deficient Zr centers in the framework, reinforces the strong coordination bonds that exist between the electron-donating groups from the π orbitals of the adsorbate and the metal. This phenomenon also affects the selectivity of gas adsorption [36].

**Table 1:** Kováts retention indices of McReynolds probes on squalane and UiO-66 columns.

Column	Retention Index, I				
	Benzene	1-Butanol	2-Pentanone	Nitropropane	Pyridine
UiO-66 PLOT	651	1408	1514	1621	1533
Squalane	650	600	620	650	709

**Table 2:** McReynolds values for UiO-66, other reported MOFs, and selected commercial stationary phases.

Column	Stationary Phase	McReynolds Constants *, Δ*I*							Ref.
		X’	Y’	Z’	U’	S’	Total	Δ*I*_Average_	
Packed MOF-5	MOF-5	−101	−33	1	−28	−23	−184	−37	[37]
Coated MIL-100 (Fe)	MIL-100 (Fe)	−45.8	−2.6	56.4	27	−104.6	−69.6	−14	[32]
Coated MIL-100 (Cr)	MIL-100 (Cr)	−32.5	97.9	44.6	43.7	−42.1	111.6	22	[32]
SPB-1	poly(dimethyl siloxane)	4	58	43	56	38	199	40	[38]
SPB-5	poly(5% diphenyl-95% dimethyl siloxane)	19	74	64	93	62	312	63	[38]
Coated MOF-CJ3	MOF-CJ3	61	169	136	242	157	765	153	[39]
SPB-50	poly(50% diphenyl-50% dimethyl siloxane)	125	175	183	268	220	971	194	[38]
SP-2340	poly(bis-cyanopropyl siloxane)	419	654	541	758	637	3009	602	[38]
UiO-66 PLOT	UiO-66	1	808	894	971	824	3498	700	Present work

* The symbols X’–Y’ indicate the probes benzene, 1-butanol, 2-pentanone, nitropropane, and pyridine, respectively.

### 2.3. Separation Performance of the UiO-66 PLOT Column

**Figure 2:**
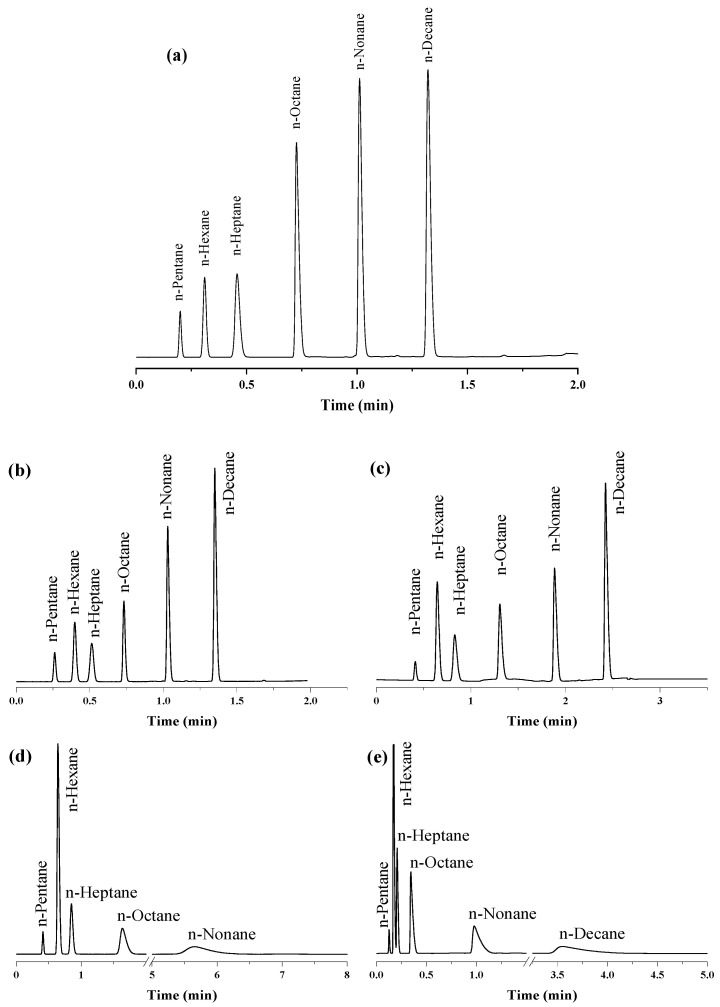
Separation of n-alkanes on UiO-66 PLOT at He flow rate of 3 mL/min with split injections at different temperature modes: (**a**) temperature program (135–250 °C, 100°/min), (**b**) temperature program (130–250 °C, 85°/min), (**c**) temperature program (120–250 °C, 50°/min), (**d**) isothermal analysis at 120 °C, (**e**) isothermal analysis at 150 °C.

The chromatographic performance of the fabricated column was evaluated using several small organic compounds. A series of six linear alkanes from *n*-pentane to *n*-decane (C_5–_C_10_) was baseline separated (R_s_ ≥ 4.36) within 1.35 min using a temperature program from 135 to 250 °C at a temperature ramp of 100°/min (Figure 2a). In addition to achieving fast and efficient separation, the temperature program also resulted in narrower peak widths of 0.02, 0.04, 0.05, 0.04, 0.04, and 0.04 min, respectively.

Several other isothermal and programmed temperature separations were also achieved to determine the effect of temperature on the separation of the chosen *n*-alkanes. As shown in Figure 2b,c, decreasing the temperature rate and the initial temperature of the programs to 130 °C and 120 °C, respectively, increased the run time and broadened the peaks while maintaining baseline separation. Likewise, isothermal analysis at 120 °C (Figure 2d) for 15 min achieved baseline peak separation (R_s_ ≥ 3.05) but caused broadening of the *n*-nonane peak, while the *n*-decane peak was not observed. The disappearance of the *n*-decane peak from the chromatogram may refer to the need for a longer run time or a higher elution temperature for the heavier *n*-decane. Raising the column temperature to 150 °C (Figure 2e) accelerated the isothermal separation process (within 4 min), with a minimum resolution of 1.44. The elution sequence of the six *n*-alkanes demonstrated the dependence of their retention on the boiling point order of the eluents and the prevailing van der Waals interactions between the n-alkanes and the hydrophobic inner surfaces of the UiO-66 cavities.

**Figure 3:**
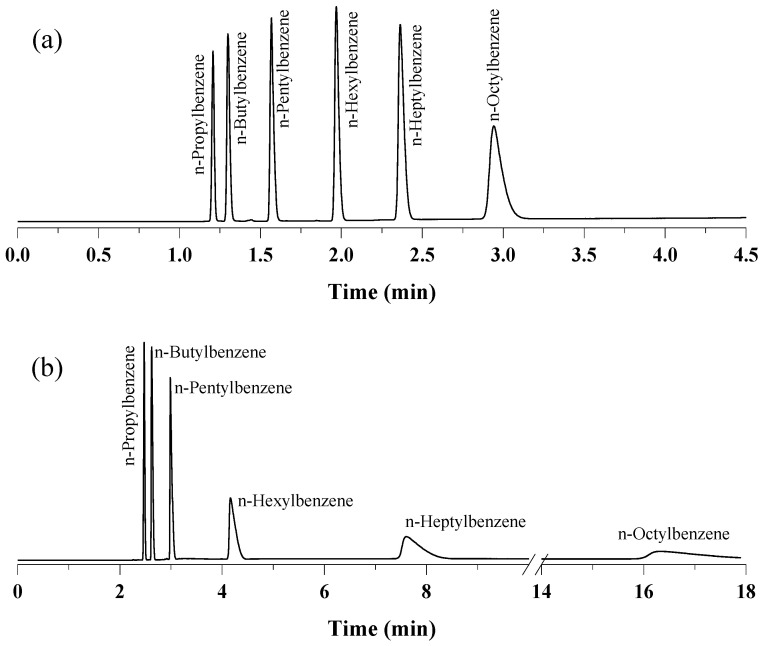
Separation of n-alkylbenzenes on UiO-66 PLOT at He flow rate of 3 mL/min with split injections at different temperature programs: (**a**) 80–250 °C, 100°/min, (**b**) 80–200 °C, 50°/min.

The separation capabilities of the prepared UiO-66 PLOT column were also investigated by injecting a mixture of six linear alkylbenzenes from *n*-propylbenzene to *n*-octylbenzene (C_3_–C_8_). As shown in Figure 3a, baseline separation (R_s_ ≥ 3.09) was achieved within 3 min of using a temperature program from 80 to 250 °C with a 100°/min ramp. Changing the temperature program to 80–200 °C at a temperature ramp of 50°/min also resulted in complete separation, but broader peaks were observed for the three heaviest homologs (i.e., C_6_–C_8_) (Figure 3b). As shown in the chromatograms, the elution order followed the increasing order of the boiling points of the components. The retention of alkylbenzenes on the UiO-66 stationary phase can be attributed to the van der Waals interactions and the π–π interactions between the hydrophobic phenyl rings on both the UiO-66 and aromatic solutes.

**Figure 4:**
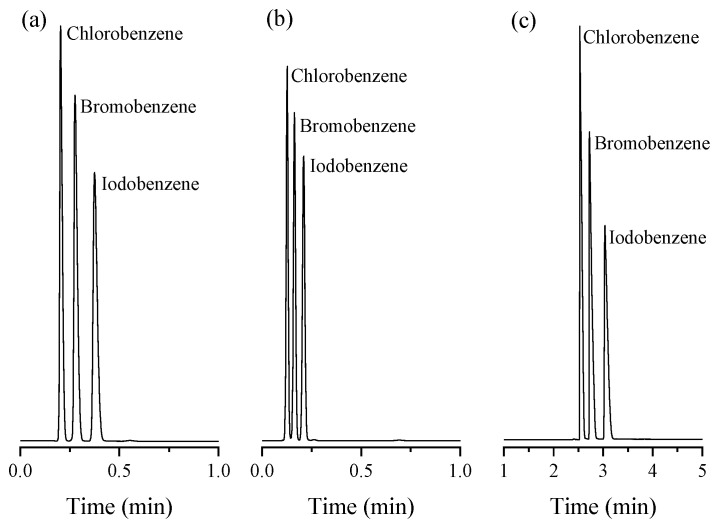
Separation of monohalobenzenes on UiO-66 PLOT at He flow rate of 3 mL/min with split injections at different temperature modes: (**a**) isothermal analysis at 200 °C, (**b**) isothermal analysis at 220 °C, (**c**) temperature program (60–180 °C, 50°/min).

The prepared column also provided fast baseline isothermal separation (R_s_ ≥ 2.63) for a mixture of monohalobenzenes (i.e., chloro, bromo, and iodobenzene) within 26 s at 200 °C (Figure 4a). The column offered theoretical plate numbers (N) of 711, 1394, and 1444 plate/m for chlorobenzene, bromobenzene, and iodobenzene, respectively. Raising the temperature to 220 °C decreased the elution time to 14 s, while maintaining total separation with a minimum resolution factor of 1.92 (Figure 4b). Additionally, the separation of the three halobenzenes was performed at lower temperatures using a temperature program between 60 and 180 °C with a ramp of 50°/min (Figure 4c). As shown, complete separation was achieved (R_s_ ≥ 2.27) within 3.2 min. It can be seen that the elution sequence followed the increasing order of the eluent boiling point. As with the alkylbenzenes, the retention of the halobenzenes can be attributed to different types of interactions, including π–π interactions between the hydrophobic phenyl rings on both UiO-66 and the halobenzenes, van der Waals interactions, and hydrogen bonds between the halogen atoms and the polar functionalities (i.e., –COOH and –OH) within the structure of UiO-66.

**Figure 5:**
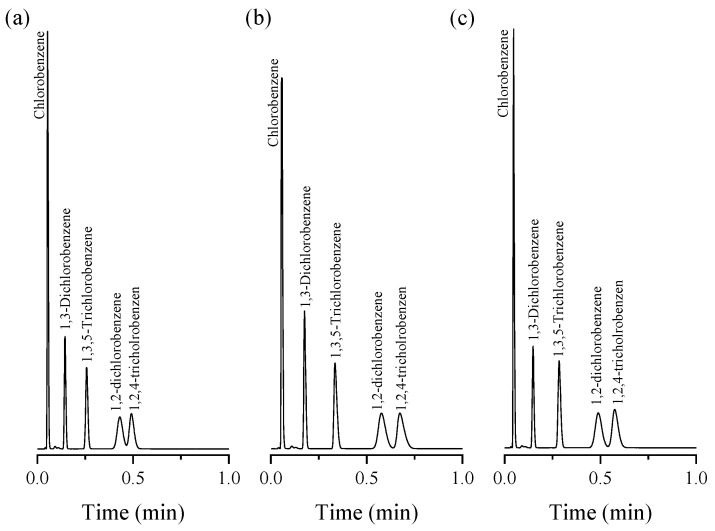
Separation of chlorobenzene isomers at different isothermal conditions; (**a**) 220 °C, flow rate of 2.5 mL/min, (**b**) 210 °C, flow rate of 2.5 mL/min, (**c**) 210 °C, flow rate of 3 mL/min, with split injections.

The ability of the UiO-66 PLOT column to separate halobenzene isomers was also assessed by injecting a chlorobenzenes mixture, including chlorobenzene (CB), 1,2- and 1,3-dichlorobenzene (1,2-/1,3-DCB), and 1,2,4- and 1,3,5-trichlorobenzene (1,2,4-/1,3,5-TCB), at 220 °C. As shown in Figure 5a, separation was achieved between structural isomers (i.e., 1,2-/1,3-DCB and 1,2,4-/1,3,5-TCB) in less than 35 s. Also, a good separation (R_s_ = 1.13) was obtained between the last two peaks (i.e., 1,2-DCB/1,2,4-TCB). Decreasing the separation temperature to 210 °C improved the resolution value to 1.37 for the separation of the 1,2-DCB/1,2,4-TCB (Figure 5b). The effect of the flow rate on the separation of the five components was evaluated by conducting the separation protocols at the same temperature (210 °C) but with an increasing flow rate from 2.5 to 3 mL/min. As shown in Figure 5c, no significant change in the peak width was noted and a slight decrease in the resolution (R_s_ = 1.34) was observed. Here, the number of theoretical plates (N) for the five peaks of the halobenzene isomers are 100, 900, 1495, 600, and 812 plate/m, respectively.

The elution of the five halobenzenes corresponded to the following increasing order: chlorobenzene (bp. 132 °C) < 1,3-dichlorobenzene (bp. 173 °C) < 1,3,5-trichlorobenzene (bp. 208 °C) < 1,2-dichlorobenzene (bp. 180 °C) < 1,2,4-trichlorobenzene (bp. 214.4 °C). The order is correlated to the boiling point of the respective components, except for 1,3,5-TCB and 1,2-DCB, which followed a reversed order. A possible explanation for this behavior might be that the meta-substituted 1,3-DCB and 1,3,5-TCB are bulkier than the ortho-substituted 1,2-DCB and 1,2,4-TCB; thus, their access to the narrow UiO-66 pores is hindered, inducing a lower retention.

In general, the window diameter of the UiO-66 cavities (5–7 Å) is similar to the critical diameter of the alkanes and benzene homologs [35]; this may explain the efficiency of separation of the UiO-66 PLOT column. Additionally, all separation chromatograms obtained in this study exhibited a very stable baseline, indicating that there was no detectable column bleed.

**Figure 6:**
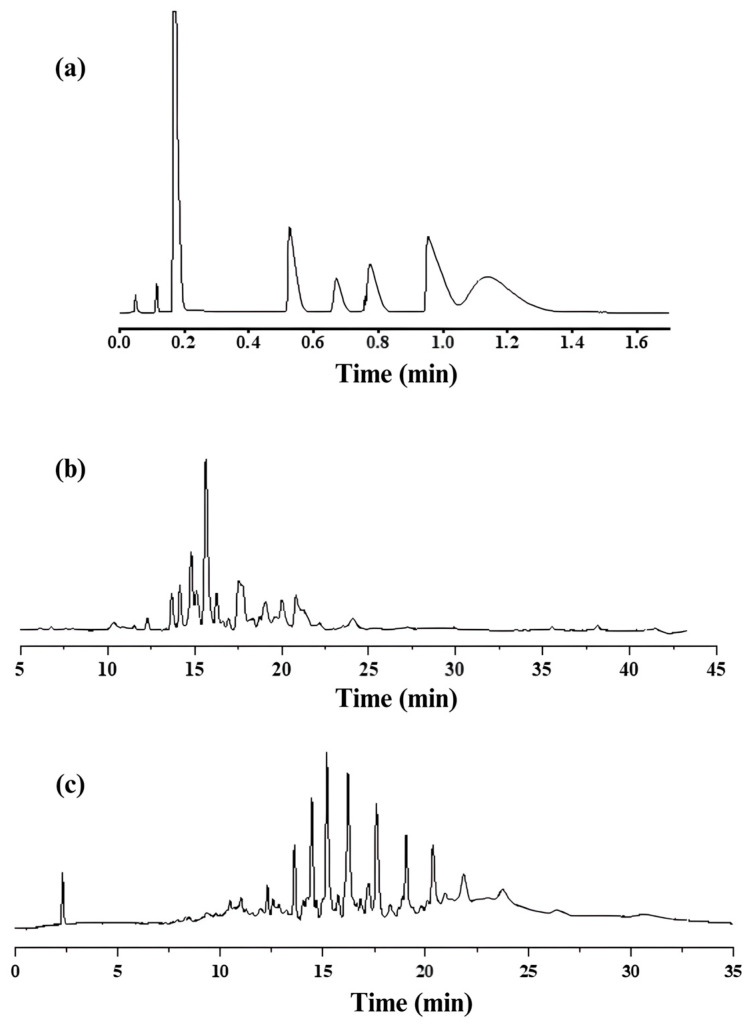
GC-FID chromatograms for analysis of (**a**) LPG sample at temperature program 60–220 °C, 20°/min with flow rate of 3 mL/min; (**b**) gasoline sample at temperature program 60–280 °C, 5°/min with flow rate of 3 mL/min; (**c**) diesel sample at temperature program 80–280 °C, 10°/min with flow rate of 3 mL/min.

To demonstrate the capability of the UiO-66 PLOT column to separate real samples, different petroleum-based samples were analyzed. Figure 6a shows the fast and good separation of the components of a sample of commercial liquified petroleum gas (LPG). Gasoline fuel was also successfully analyzed as shown in Figure 6b. In addition, the ability of the UiO-66 PLOT column to separate the main components of a diesel sample can be seen from the GC-FID chromatogram in Figure 6c.

### 2.4. Exceptional Thermal Stability of UiO-66 PLOT Columns

**Figure 7:**
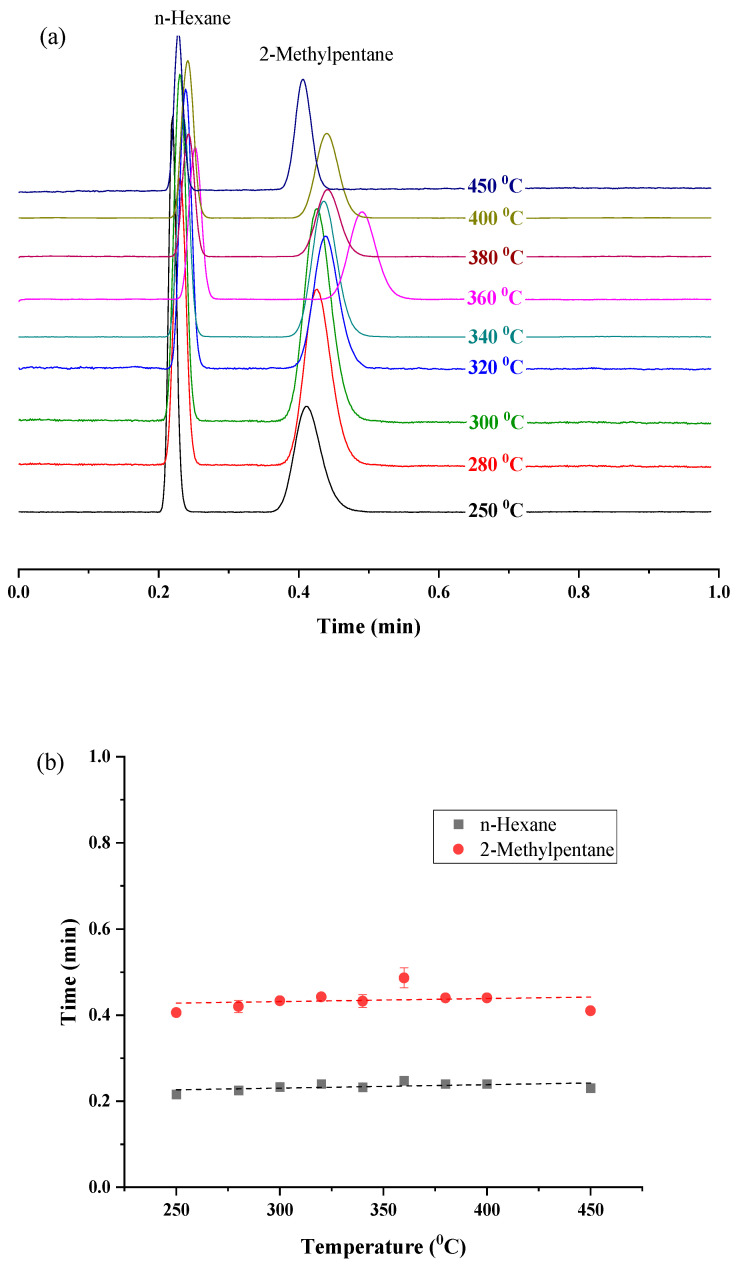
(**a**) GC chromatograms for the separation of hexane and 2-methylpentane isomers isothermally at 150 °C with a flow rate of 2.5 mL/min, after heating the UiO-66 column at different temperatures; (**b**) effect of heating the columns at elevated temperatures on the retention times of the two isomers.

For the determination of the maximum operating temperature of the prepared UiO-66 PLOT columns, the column was heated for one hour at several temperatures in the range of 250–450 °C using the GC-FID system. After each heating cycle, a mixture of hexane and 2-methylpentane isomers was analyzed isothermally at 150 °C (Figure 7a).

It can be seen from Figure 7b that the relation between the heating temperature and the retention times of the solutes is mostly constant. This steady performance reflects the exceptional thermal stability of the prepared columns in the studied temperature range. Moreover, the stable baseline of the chromatograms (Figure 7a) indicates that there was no bleeding of the stationary phase. This also proves that the prepared MOF stationary phase is firmly attached to the fused silica surface. The slight deviation in the retention time of 2-methylpentane after heating the column at 360 °C and 450 °C, compared to the retention time at 150 °C (before heating the column at elevated temperatures), is expressed as relative percent differences (%RPD) of 24.56% and 30.63%, respectively. Both values are within the acceptable range for %RPD (≤30%) according to the United States Environmental Protection Agency (EPA) [40].

**Figure 8:**
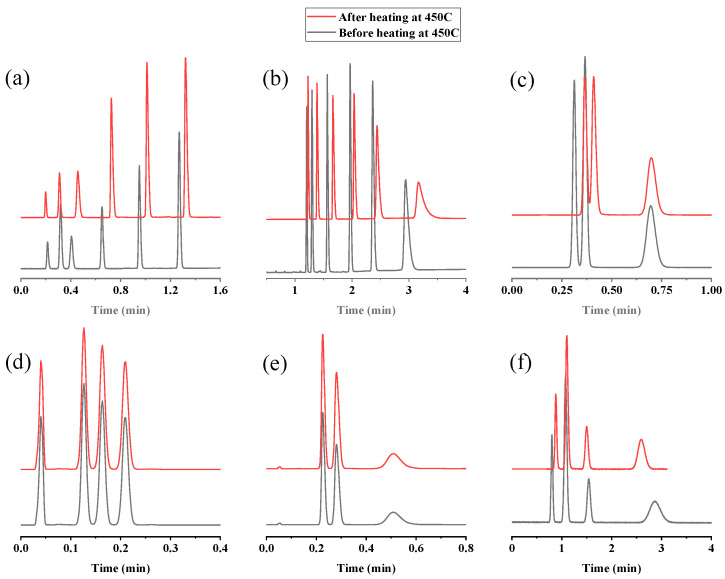
GC chromatograms for the separation of different mixtures after heating the UiO-66 PLOT column at 450 °C: (**a**) separation of n-alkanes (C_5_–C_10_) at 135–250 °C, 10°/min, flow rate 3 mL/min; (**b**) separation of n-alkylbenzenes (C_3_–C_8_) at 80–250 °C, 100°/min, flow rate 3 mL/min; (**c**) separation of iso-, n-, and tert-butylbenzene at 200 °C, flow rate 2.5 mL/min; (**d**) separation of chloro, bromo, and iodobenzene at 220 °C, flow rate 3 mL/min; (**e**) separation of p-, m-, and o-chlorotoluene at 200 °C, flow rate 3 mL/min; (**f**) separation of p-xylene, m-xylene, ethylbenzene, and o-xylene at 150 °C, flow rate 3 mL/min.

For further confirmation, different mixtures, including n-alkanes, n-alkylbenzenes, halobenzenes, butylbenzenes isomers, chlorotoluene isomers, and xylene isomers, were re-injected at their optimum separation conditions after heating the column at 450 °C (Figure 8). Table 3 represents the high precision (expressed in relative percent difference (%RPD)) of the retention times of the analyzed solutes before and after heating the column to 450 °C. As shown, the %RPD values for all analytes range from 0.00% to 14.93%. However, for 2-methylpentane, it is 30.63%, which is still aligned with the accepted values (≤30%) [40].

The superior thermal stability of UiO-66 PLOT columns can be attributed to the two following aspects. First, the high thermal stability, up to 540 °C, of the UiO-66 structure. Second, the high thermal stability of COOH-terminated SAMs which were formed by the silanization process of the fused silica surface.

Table 4 compares the maximum operating temperatures for UiO-66 columns with those of Agilent’s high-temperature GC columns [41], which are engineered from special high-temperature polyimide-coated fused-silica tubing. Obviously, the UiO-66 PLOT column has the highest maximum temperature (450 °C) compared to the listed common commercial columns. It should also be noted that according to previous studies, UiO-66 is thermally stable till 540 °C, but our investigations were limited to 450 °C, which was the highest temperature allowed by the GC instrument. The high thermal stability of the UiO-66 PLOT column enabled it to analyze a variety of petroleum samples (liquefied petroleum gas, gasoline, diesel), as detailed in Section 2.3. This capability may open up broad prospects for its use in analyzing complex mixtures with high boiling points, such as petroleum products, natural extracts, waxes, and surfactants. It also encourages its application in GC-MS technology.

## 3. Materials and Methods

### 3.1. Chemicals and Materials

All chemicals used within this work were either analytical or chromatographic grade and were used without further treatment. Ethanol ≥ 99.8%, *N*,*N*-dimethylformamide ≥ 99.8% (DMF), glacial acetic acid ≥ 99.7%, benzene-1,4-dicarboxylic acid (BDC) ≥ 98.0%, bromobenzene ≥ 99.5%, iodobenzene ≥ 97.5%, *n*-pentylbenzene 1,2-dichlorobenzene *n*-heptylbenzene ≥ 97.5%, and *n*-alkanes (C_5_–C_12_) ≥ 97.0% were all purchased from Sigma-Aldrich (St. Louis, MO, USA). Sodium hydroxide ≥ 98.0%, 1,2-dichlorobenzene 98.5%, and 1,3-dichlorobenzene 99.0% were obtained from Merck (Darmstadt, Germany). Zirconium (IV) chloride ≥ 97.5%, potassium permanganate ≥ 99.0%, *n*-hexylbenzene ≥ 97.5%, *n*-propylbenzene ≥ 97.5%, *n*-butylbenzene ≥ 98.5%, and *n*-octylbenzene ≥ 98.5% were obtained from Acros (Geel, Belgium). 3-Aminopropyltriethoxysilane (APTES) ≥ 98.0%, chlorobenzene ≥ 99.5%, and 1,3,5-trichlorobenzene ≥ 98.0% were purchased from Alfa Aesar (Ward Hill, MA, USA). Glutaraldehyde (25% in H_2_O) and 1,2,4-trichlorobenzene ≥ 99.0% were obtained from Fluka (Buchs, Switzerland). The ultrapure water used in the preparation procedures was obtained using a Millipore Milli-Q water system (St. Louis, MO, USA). The high-purity grade gases, namely, helium, air, and hydrogen (99.9999%), were obtained from SIGAS (Riyadh, Saudi Arabia). Untreated polyimide-coated fused-silica capillary (320 μm i.d.) was purchased from Polymicro Technologies (Phoenix, AZ, USA).

### 3.2. Instrumentation

Chromatography was performed on a Thermo Scientific Trace GC Ultra system equipped with a flame ionization detector (FID) and a split/splitless injector (Austin, TX, USA). The temperature of the FID detector and the inlet were set to 250 °C. Helium was used as carrier gas. The samples were manually injected using a 10 µL Hamilton 701N syringe. Data acquisition and processing were controlled by Xcalibur 3.0 software. The morphology of the inner walls of the bare and coated capillaries was characterized using a JEOL (JSM-7610F) scanning electron microscope coupled with an energy-dispersive X-ray system (SEM–EDX) (Tokyo, Japan) operating at 15 kV with platinum coating.

### 3.3. Preparation of the Carboxyl-Terminated Capillary (SAM)

**Figure 9:**
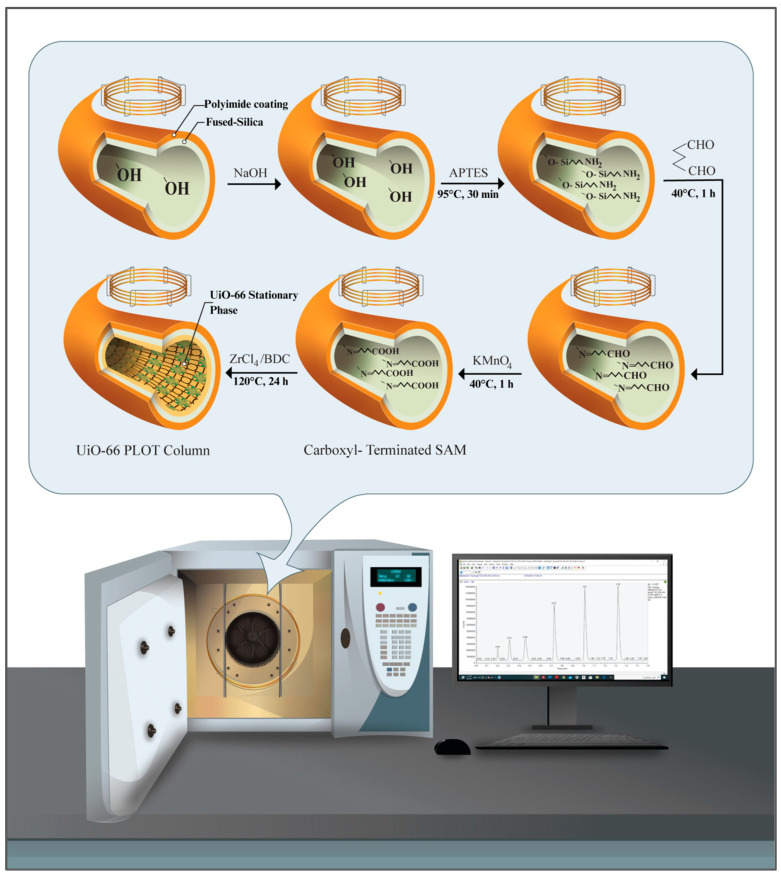
Schematic illustration for the preparation steps of the UiO-66-bonded PLOT columns.

Figure 9 schematically summarizes the preparation of UiO-66-bonded PLOT columns. The COOH-terminated fused-silica capillaries were prepared as reported previously, but with slight modifications [26].

Prior to the functionalization process, the silanol groups on the bare capillary were activated using sequential NaOH-etching and HCl-leaching treatments. For this purpose, the capillary (2.5 m length, 320 µm i.d.) was sequentially rinsed with 1 M NaOH for at least 4 h, with ultrapure water to pH 7, and 0.1 M HCl for 30 min, and again with water to pH 7. The capillary was subsequently dried by heating at 100 °C under a constant helium gas flow for 4 h. The amino-terminated SAM was then prepared by filling the dried fused-silica capillary with an aqueous solution of APTES (10%, *v*/*v*), sealing the column at both ends and heating it in an oven at 95 °C for 30 min. The APTES treatment process was repeated four times to ensure sufficient coating of the amino groups on the capillary inner wall. The APTES-modified capillary was then rinsed with water to remove any residual APTES solution. Next, the amino groups were converted to aldehydes by injecting an alkaline solution of glutaraldehyde (2%, pH 8) into the capillary and allowing the reaction to proceed for 1 h at 40 °C. The capillary was then rinsed with water to remove the residual glutaraldehyde solution. The aldehydes were oxidized to carboxyl groups by injecting an aqueous solution of 0.1 M KMnO_4_ into the capillary and allowing the reaction to proceed for 1 h at 40 °C. The carboxyl-terminated capillary was then rinsed with water for 3 h and ethanol for 1 h before being dried overnight at 100 °C under a constant flow of helium. The solutions were injected into the column using a syringe pump at a constant flow rate of 0.3 mL/h.

### 3.4. Preparation of the UiO-66-Bonded PLOT Column

In situ growth of the UiO-66 polycrystalline film was achieved by filling the dried COOH-terminated capillary with the MOF precursor solution, sealing both ends of the column, and heating it at 120 °C for 24 h. The UiO-66 precursor solution (10 mM) was prepared by dissolving ZrCl_4_ and BDC with a 1:1 molar ratio in DMF/AcOH [42]. The capillary was then rinsed with ethanol for 3 h before being thermally conditioned as follows: an initial step at 40 °C for 30 min and a temperature ramp from 40 °C to 120 °C at a rate of 1°/min were conducted before holding at 120 °C for 120 min. A second temperature ramp from 120 °C to 200 °C was applied at the same rate before the program was held for 240 min at the final temperature. Small pieces of the prepared UiO-66 column were then used to obtain SEM–EDX micrographs to verify the formation of the UiO-66 film.

### 3.5. Determination of the McReynolds Constants

Determining the associated McReynolds constants for a column stationary phase is an effective, albeit traditional, way to assess the polarity and selectivity of the stationary phase for specific functional groups. Here, the polarity of the MOF layer phase was evaluated relative to a reference stationary phase, which is usually non-polar squalane (2,6,10,15,19,23-hexamethyltetracosane, C_30_H_62_). These constants are based on the retention behavior of five probe molecules, namely, benzene, *n*-butanol, pentan-2-one, nitropropane, and pyridine. Each probe reflects a specific intermolecular interaction with the stationary phase. The benzene parameter, X’, is related to the electron density for aromatic and olefinic hydrocarbons. n-Butanol, Y’, reflects the probe proton donor and proton acceptor capabilities. 2-Pentanone, Z’, describes the associated proton acceptor interactions. The parameters of nitropropane, U’, and pyridine, S’, are related to the dipole interactions and strong proton acceptor interactions, respectively. Thus, it is possible to classify the chromatographic stationary phases according to their polarity using the associated McReynolds numbers (Δ*I*). The average polarity can be calculated as the sum of the Δ*I* values of the five probes. The stationary phases with McReynolds values lower than 100 are considered non-polar, whereas the materials having values between 100 and 400 are described as moderately polar. High polar phases usually exhibit McReynolds constants higher than 400 [43,44].

In this study, a squalane (2,6,10,15,19,23-hexamethyltetracosane, C_30_H_62_) column was used as the reference non-polar stationary phase. All probe molecules were injected at 100 °C on the reference column and at 250 °C on the UiO-66 column, except for benzene, which was injected at 100 °C. The McReynolds constants (Δ*I*) were calculated as the difference between the Kováts retention indices of each probe on the considered column and the reference one.

The Kováts retention indices (I_X_) were calculated using the following equation:

(1) $$I_{X}=100n+100\frac{\log\left(t_{R(x)}\right)-\log\left(t_{R(n)}\right)}{\log\left(t_{R\left(n+1\right)}\right)-\log\left(t_{R(n)}\right)}$$

where t_R(n)_ and t_R(n+1)_ are the retention times of the reference *n*-alkanes eluted immediately before and after a given solute “X”. t_R(X)_ is the retention time of compound “X”.

## 4. Conclusions

In this study, a UiO-66 metal–organic framework stationary phase was successfully grown on the inner wall of a COOH-terminated fused-silica capillary using a straightforward, one-step in situ solvothermal procedure. The growth of the stationary phase film was confirmed using SEM–EDX analysis. SEM micrographs showed homogenous, continuous UiO-66 coating on the surface of the prepared columns. The average polarity calculated using McReynolds constants reflected the high polar nature of the bonded UiO-66 stationary phase. The fabricated UiO-66 PLOT columns were capable of the efficient, fast, and complete separation of various small organic compounds, including *n*-alkanes, *n*-alkylbenzenes, monohalobenzenes, and polysubstituted halobenzene isomers. The very stable baseline of the chromatograms, the repeatability of the results, and the absence of any column bleeding confirmed the stability of the prepared columns. It was also able to efficiently analyze various real petroleum-based samples. The thermal stability study of the UiO-66 PLOT column proved that the column performance was not significantly affected even after heating above 400 °C, which means it had notably high thermal stability. The findings of the present study open the door for widespread integration of the UiO-66 PLOT column into gas chromatography–mass spectrometry (GC–MS) systems for efficient GC separation of complex mixtures such as petroleum fractions and natural extracts.

## Figures and Tables

**Table 3:** Comparison of separation performance of UiO-66 PLOT column before and after heating at 450 °C in terms of retention times of the analytes.

Analyte ^a^	t_R_, min ^b^ Before Heating at 450 °C	t_R_, min ^b^ After Heating at 450 °C	%RPD ^c^
n-Hexane	0.28	0.24	13.59
2-methylpntane	0.64	0.47	30.63
n-pentane	0.22	0.20	12.05
n-Hexane	0.32	0.30	6.45
n-Heptane	0.41	0.43	3.59
n-Octane	0.65	0.69	5.97
n-Nonane	0.95	0.99	3.62
n-Decane	1.27	1.29	1.56
n-Probylbenzene	1.21	1.23	1.64
n-butylbenzene	1.30	1.39	6.69
n-Pentylbenzene	1.57	1.67	6.17
n-Hexylbenzene	1.97	2.05	3.99
n-Heptylbenzene	2.36	2.45	3.74
n-Octylbenzene	2.94	3.20	8.31
iso-butylbenzene	0.31	0.36	14.93
n-Butylbenzene	0.37	0.41	11.13
tert-Butylbenzene	0.70	0.70	0.72
Chlorobenzene	0.12	0.12	0.00
Bromobenzene	0.16	0.16	0.00
Iodobenzene	0.2	0.21	4.80
p-Chlorotoluene	0.23	0.23	2.20
m-Chlorotoluene	0.28	0.29	0.00
o-Chlorotoluene	0.51	0.49	4.00
p-Xylene	0.80	0.88	9.52
m-Xylene	1.08	1.10	1.83
Ethylbenzene	1.54	1.50	2.63
o-Xylene	2.86	2.59	9.91

^a^ Separated at their optimum conditions that are described in Figure 8. ^b^ Average of 3 replicates. ^c^ Relative percent difference.

**Table 4:** Specifications of UiO-66 PLOT and Agilent’s high-temperature GC columns.

Stationary Phase	Polarity	Common Name	Maximum Temperature, °C
100% Dimethylpolysiloxane	Non-polar	DB-1ht	400
(5%-Phenyl)-methylpolysiloxane	Non-polar	DB-5ht	400
(50%-Phenyl)-methylpolysiloxane	Mid-polarity	DB-17ht	365
UiO-66	High polar	UiO-66-bonded PLOT	450

## Data Availability

Data are contained within the article.

## References

[1] Barry E.F., Grob R.L. (2007). Columns for Gas Chromatography.

[2] Dorman F.L., Dawes P., Poole C.F. (2012). Gas Chromatography.

[3] Yaghi O.M., Li G., Li H. (1995). Selective binding and removal of guests in a microporous metal–organic framework. Nature.

[4] Hoffmann F., Fröba M., Kaskel S. (2016). The Chemistry of Metal-Organic Frameworks.

[5] Zhao X., Wang Y., Li D.S., Bu X., Feng P. (2018). Metal–organic frameworks for separation. Adv. Mater..

[6] Almáši M. (2021). A review on state of art and perspectives of Metal-Organic frameworks (MOFs) in the fight against coronavirus SARS-CoV-2. J. Coord. Chem..

[7] Dey C., Kundu T., Biswal B.P., Mallick A., Banerjee R. (2014). Crystalline metal-Organic frameworks (MOFs): Synthesis, structure and function. Acta Crystallogr. Sect. B Struct. Sci. Cryst. Eng. Mater..

[8] Stock N., Biswas S. (2012). Synthesis of metal-organic frameworks (MOFs): Routes to various MOF topologies, morphologies, and composites. Chem. Rev..

[9] Valizadeh B., Nguyen T.N., Stylianou K.C. (2018). Shape engineering of metal–organic frameworks. Polyhedron.

[10] Siemensmeyer K., Peeples C.A., Tholen P., Schmitt F.-J., Çoşut B., Hanna G., Yücesan G. (2020). Phosphonate Metal–Organic Frameworks: A Novel Family of Semiconductors. Adv. Mater..

[11] Leelasree T., Goel S., Aggarwal H. (2022). MOF-Based Dual Sensor for the Electrochemical and Fluorescence Detection of Nicotine. ACS Appl. Nano Mater..

[12] Király N., Capková D., Gyepes R., Vargová N., Kazda T., Bednarčík J., Yudina D., Zelenka T., Čudek P., Zeleňák V. (2023). Sr(II) and Ba(II) Alkaline Earth Metal–Organic Frameworks (AE-MOFs) for Selective Gas Adsorption, Energy Storage, and Environmental Application. Nanomaterials.

[13] Ye Y., Xian S., Cui H., Tan K., Gong L., Liang B., Pham T., Pandey H., Krishna R., Lan P.C. (2022). Metal–Organic Framework Based Hydrogen-Bonding Nanotrap for Efficient Acetylene Storage and Separation. J. Am. Chem. Soc..

[14] Kuzharov A.A., Gritsai M.A., Butova V.V., Soldatov M.A., Polyakov V.A., Rud P.A., Rusalev Y.V., Kubrin S.P., Roldugin V.A., Trigub A.L. (2022). One-step electrochemical synthesis of γ-Fe_2_O_3_@MIL-88a magnetic composite for heterogeneous Fenton-like catalysis. Ceram. Int..

[15] Li H., Li C., Wu Y., Wang C., Guo T., Zhang J., Sun L. (2021). Cross-linked γ-cyclodextrin metal-organic framework—A new stationary phase for the separations of benzene series and polycyclic aromatic hydrocarbons. Microchim Acta.

[16] Gao B., Huang M., Zhang Z., Yang Q., Su B., Yang Y., Ren Q., Bao Z. (2019). Hybridization of metal–organic framework and monodisperse spherical silica for chromatographic separation of xylene isomers. Chin. J. Chem. Eng..

[17] Yang S., Xie H., Zhu H., Zhang L., Zhou Y., Zhang H., Zhao Z. (2018). Highly effective hydrogen isotope separation by cryogenic gas chromatography in a new stationary phase material MnCl2@CPL-1@γ-Al_2_O_3_. Int. J. Hydrogen Energy.

[18] Li Z., Mao Z., Zhou W., Chen Z. (2020). Incorporation of homochiral metal-organic cage into ionic liquid based monolithic column for capillary electrochromatography. Anal. Chim. Acta.

[19] Yusuf K., Badjah-Hadj-Ahmed A.Y., Aqel A., Alothman Z.A. (2016). Monolithic metal-organic framework MIL-53(Al)-polymethacrylate composite column for the reversed-phase capillary liquid chromatography separation of small aromatics. J. Sep. Sci..

[20] Wu Y.-Y., Yang C.-X., Yan X.-P. (2015). An in situ growth approach to the fabrication of zeolite imidazolate framework-90 bonded capillary column for gas chromatography separation. Analyst.

[21] Gu Z.Y., Jiang J.Q., Yan X.P. (2011). Fabrication of isoreticular metal-organic framework coated capillary columns for high-resolution gas chromatographic separation of persistent organic pollutants. Anal. Chem..

[22] Shekhah O., Liu J., Fischer R.A., Wöll C. (2011). MOF thin films: Existing and future applications. Chem. Soc. Rev..

[23] Münch A.S., Seidel J., Obst A., Weber E., Mertens F.O.R.L. (2011). High-separation performance of chromatographic capillaries coated with MOF-5 by the controlled SBU approach. Chem. A Eur. J..

[24] Gu Z.G., Zhang J. (2019). Epitaxial growth and applications of oriented metal–organic framework thin films. Coord. Chem. Rev..

[25] Tang P., Bao T., Chen Z. (2016). Novel Zn-based MOFs stationary phase with large pores for capillary electrochromatography. Electrophoresis.

[26] Bao T., Zhang J., Zhang W., Chen Z. (2015). Growth of metal–organic framework HKUST-1 in capillary using liquid-phase epitaxy for open-tubular capillary electrochromatography and capillary liquid chromatography. J. Chromatogr. A.

[27] Pan C., Wang W., Chen X. (2016). In situ rapid preparation of homochiral metal-organic framework coated column for open tubular capillary electrochromatography. J. Chromatogr. A.

[28] Xu Y.Y., Lv W.J., Ren C.L., Niu X.Y., Chen H.L., Chen X.G. (2018). In situ preparation of multilayer coated capillary column with HKUST-1 for separation of neutral small organic molecules by open tubular capillary electrochromatography. J. Chromatogr. A.

[29] Chen B., Liang C., Yang J., Contreras D.S., Clancy Y.L., Lobkovsky E.B., Yaghi O.M., Dai S. (2006). A microporous metal-organic framework for gas-chromatographic separation of alkanes. Angew. Chemie Int. Ed..

[30] Chang N., Gu Z.Y., Yan X.P. (2010). Zeolitic imidazolate framework-8 nanocrystal coated capillary for molecular sieving of branched alkanes from linear alkanes along with high-resolution chromatographic separation of linear alkanes. J. Am. Chem. Soc..

[31] Münch A.S., Mertens F.O.R.L. (2012). HKUST-1 as an open metal site gas chromatographic stationary phase—Capillary preparation, separation of small hydrocarbons and electron donating compounds, determination of thermodynamic data. J. Mater. Chem..

[32] Fan L., Yan X.P. (2012). Evaluation of isostructural metal-organic frameworks coated capillary columns for the gas chromatographic separation of alkane isomers. Talanta.

[33] Xie S.M., Zhang M., Fei Z.X., Yuan L.M. (2014). Experimental comparison of chiral metal-organic framework used as stationary phase in chromatography. J. Chromatogr. A.

[34] Cavka J.H., Jakobsen S., Olsbye U., Guillou N., Lamberti C., Bordiga S., Lillerud K.P. (2008). A new zirconium inorganic building brick forming metal organic frameworks with exceptional stability. J. Am. Chem. Soc..

[35] Chang N., Yan X.P. (2012). Exploring reverse shape selectivity and molecular sieving effect of metal-organic framework UIO-66 coated capillary column for gas chromatographic separation. J. Chromatogr. A.

[36] Sladekova K., Campbell C., Grant C., Fletcher A.J., Gomes J.R.B., Jorge M. (2019). The effect of atomic point charges on adsorption isotherms of CO_2_ and water in metal organic frameworks. Adsorption.

[37] Gu Z., Jiang D., Wang H., Cui X., Yan X. (2010). Adsorption and separation of xylene isomers and ethylbenzene on two Zn-terephthalate metal-organic frameworks. J. Phys. Chem. C.

[38] Supelco-Inc (1997). The retention index system in gas chromatography: McReynolds constants. Supelco GC Bull..

[39] Fang Z.L., Zheng S.R., Tan J.B., Cai S.L., Fan J., Yan X., Zhang W.G. (2013). Tubular metal-organic framework-based capillary gas chromatography column for separation of alkanes and aromatic positional isomers. J. Chromatogr. A.

[40] Morgan M.K., Sheldon L.S., Croghan C.W., Chuang J.C., Lordo R.A., Wilson N.K., Lyu C., Brinkman M., Morse N., Chou Y.L. (2005). A Pilot Study of Children’s Total Exposure to Persistent Pesticides and Other Persistent Organic Pollutants (CTEPP).

[41] (2020). Agilent, Performance and Durability for High-Temperature GC. https://www.agilent.com/cs/library/brochures/brochure-high-temp-gc-columns-5994-1384en-agilent.pdf.

[42] Miyamoto M., Kohmura S., Iwatsuka H., Oumi Y., Uemiya S. (2015). In situ solvothermal growth of highly oriented Zr-based metal organic framework UiO-66 film with monocrystalline layer. CrystEngComm.

[43] Barry E.F., Grob R.L., Barry E.F. (2004). Modern Practice of Gas Chromatography.

[44] Sun T., Tian L., Li J., Qi M., Fu R., Huang X. (2013). Dithienyl benzothiadiazole derivatives: A new type of stationary phases for capillary gas chromatography. J. Chromatogr. A.

